# Identifying, Prioritizing and Visually Mapping Barriers to Injury Care in Rwanda: A Multi-disciplinary Stakeholder Exercise

**DOI:** 10.1007/s00268-020-05571-6

**Published:** 2020-05-21

**Authors:** Maria Lisa Odland, John Whitaker, Dmitri Nepogodiev, Carolyn Achieng’ Aling’, Irene Bagahirwa, Theophile Dushime, Darius Erlangga, Christophe Mpirimbanyi, Severien Muneza, Menelas Nkeshimana, Martin Nyundo, Christian Umuhoza, Eric Uwitonze, Jill Steans, Alison Rushton, Antonio Belli, Jean Claude Byiringiro, Abebe Bekele, Justine Davies

**Affiliations:** 1 Institute of Applied Health Research, University of Birmingham, Birmingham, UK; 2 Faculty of Life Sciences and Medicine, King’s Centre for Global Health and Health Partnerships, King’s College London, Room 2.13, Global Health Offices, Weston Education Centre, Cutcombe Road, London, SE5 9RJ UK; 3 Academic Department of Military Surgery and Trauma, Royal Centre for Defence Medicine, Birmingham, UK; 4 National Institute for Health Research, Global Health Research Unit on Global Surgery, Institute of Translational Medicine, University of Birmingham, Birmingham, UK; 5 King Faisal Hospital, Kigali, Rwanda; 6 Rwanda Biomedical Centre, Kigali, Rwanda; 7 SAMU Division, Ministry of Health, Kigali, Rwanda; 8 Warwick Medical School, Population Evidence and Technologies, University of Warwick, Coventry, UK; 9 University of Rwanda College of Medicine and Health Sciences, Kigali, Rwanda; 10 University Teaching Hospital of Kigali, Kigali, Rwanda; 11 Department of Political Science and International Studies, School of Government and Society, University of Birmingham, Birmingham, UK; 12 School of Sport, Exercise and Rehabilitation Sciences, University of Birmingham, Birmingham, UK; 13 College of Medicine and Dental Sciences, NIHR Surgical Reconstruction and Microbiology Research Centre, University of Birmingham, Birmingham, UK; 14 University of Global Health Equity, Kigali, Rwanda; 15 Faculty of Health Sciences, Medical Research Council/Wits University Rural Public Health and Health Transitions Research Unit, University of Witwatersrand, Johannesburg, Gauteng South Africa

## Abstract

**Background:**

Whilst injuries are a major cause of disability and death worldwide, a large proportion of people in low- and middle-income countries lack timely access to injury care. Barriers to accessing care from the point of injury to return to function have not been delineated.

**Methods:**

A two-day workshop was held in Kigali, Rwanda in May 2019 with representation from health providers, academia, and government. A four delays model (delays to seeking, reaching, receiving, and remaining in care) was applied to injury care. Participants identified barriers at each delay and graded, through consensus, their relative importance. Following an iterative voting process, the four highest priority barriers were identified. Based on workshop findings and a scoping review, a map was created to visually represent injury care access as a complex health-system problem.

**Results:**

Initially, 42 barriers were identified by the 34 participants. 19 barriers across all four delays were assigned high priority; highest-priority barriers were “*Training and retention of specialist staff*”, “*Health education/awareness of injury severity*”, “*Geographical coverage of referral trauma centres*”, and “*Lack of protocol for bypass to referral centres*”. The literature review identified evidence relating to 14 of 19 high-priority barriers. Most barriers were mapped to more than one of the four delays, visually represented in a complex health-system map.

**Conclusion:**

Overcoming barriers to ensure access to quality injury care requires a multifaceted approach which considers the whole patient journey from injury to rehabilitation. Our results can guide researchers and policymakers planning future interventions.

## Introduction

Each year, one billion people sustain injuries requiring health care. Injury is a leading cause of disability and associated with over five million deaths each year [1]. Injuries account for more deaths that tuberculosis, malaria, and HIV combined, and 90% of injury deaths occur in low- and middle-income countries (LMICs) [2]. Road traffic collisions (RTC) may be the third leading global cause of death by 2030 [3]. Halving the number of global deaths and injuries due to RTCs is a key Sustainable Development Goal (SDG 3.6) [4].

Rwanda has one of the highest incidence of injuries in the world [5] and has committed to reduce morbidity and mortality due to injuries [6]. Nevertheless, in 2012, 22% of all deaths in Rwanda’s capital Kigali were from injury, with RTCs the most common mechanism [7]. In 2017, 10% of DALYS and 9% of deaths were injury related [8].

The three delays framework was developed to understand factors driving avoidable maternal deaths. It has been widely adopted in research on barriers in access to care [9]. The delays are: 1. delays in seeking care; 2. delays in reaching care; and 3. delays in receiving quality health care at a facility [10]. The framework has also been used to show delays in accessing injury care are implicated in up to 36% of injury deaths [11, 12]. Much injury care research in LMICs has focused on delay three; assessing and improving care provision in facilities. This neglects many injured people that never reach a facility, potentially 40% of avoidable mortality [11]. We adapted the three delays model, by including a fourth delay, remaining in care, distinguishing between initial receipt of emergency care and ongoing care provided as follow-up or rehabilitation [13]. This study aimed to use this four delay framework to describe delays and identify and prioritise barriers to accessing quality injury care in Rwanda [11, 12] and to visually represent the complex inter-relationships between them.

## Methods

### Setting

Rwanda is a small landlocked country in east-Africa with a low Human Development Index (HDI), ranking 158 of 189 countries [14]. Following significant economic growth since the 1994 Genocide against Tutsis, the health system has experienced major improvements. Initiatives include a national health insurance policy, performance-based financing of health programmes, and village community health workers [15, 16]. Despite improvements, health care investment in Rwanda remains insufficient [14, 17]. The Rwandan government has committed to reducing injury morbidity and mortality [6].

### Stakeholder workshop

A national stakeholder concept mapping workshop was held over 2 days in Kigali, May 2019, bringing together multi-sectoral participants involved in injury care in Rwanda. Through this workshop, this study aimed to:

1. Identify barriers in access to injury care in Rwanda.
2. Prioritize identified barriers for future research and intervention.
3. Schematically map identified barriers to the four delays framework.
4. Scope existing literature for injury care studies in Rwanda and relate findings to the workshop identified barriers.

### Participants

Participants were purposively invited from a broad range of professional backgrounds, with expertize to understand barriers to quality care from point of injury to return to optimal function. Invitations were sent to; community health providers; police, fire and rescue; telecommunications providers; prehospital care providers (Emergency Medical Services (EMS) Division/SAMU (Service d’Aide Médicale d’Urgence); secondary care injury-care providers; government ministry representatives, including ministry of health; medical students; information and technology representatives; injury and disability researchers; physiotherapists; health insurance providers; and international Rwandan-based NGOs.

### Identifying and prioritising barriers

The workshop began with an introduction to the four delays framework and an update on injury care and developments in Rwanda. Participants were divided into four groups, each focused on one conceptual delay to injury care, based on their interests and expertize.

First, groups brainstormed barriers at each of their assigned delays. If identified barriers were thought to affect additional delays, this was discussed. Second, participants ranked barriers into roughly equal groups of high, medium, and low priority based upon their impact and feasibility of addressing them with interventions. After each group discussion, findings were presented to the whole workshop. Questions and wider discussion followed with opportunity to adjust findings based on consensus.

Third, consensus on the highest four priority barriers across all delays was achieved through sequential smartphone voting using menti.com™ application [18]. Three rounds of anonymous voting were undertaken. In round one, each participant was asked to indicate their top four out of the all barriers ranked as high priority. Those with ≤5% of votes were removed. In round two, participants again selected their four highest priority barriers. If four barriers were clearly forerunners, these were to be selected and voting stopped. If fewer than four barriers were clear forerunners, those that were clear high priorities were removed and participants asked to vote on the remainder of the barriers. Participants debated results between voting stages and justified their choices.

### Scoping literature search

A scoping review searched PubMed in July 2019 for published studies relating to barriers to injury care in Rwanda. Broad search strings were [Rwanda AND (Trauma OR Injury)], (Rwanda AND delays), and (Rwanda AND barriers). There were no defined year limits or language restrictions for publications. A single author (JW) screened the articles and extracted data. Any articles of any study type that reported evidence on barriers to access to care were eligible for inclusion. Available published evidence from within the Rwandan health system was tabulated against each identified barrier.

### Analysis

In order to schematically represent barriers to accessing injury care as a complex health-system problem, the barriers proposed at the workshop were synthesized into overarching categories by authors based on established health system frameworks [19, 20]. These were also mapped to their respective delay, illustrating where they impact access to injury care. A visual map was created combining workshop discussion results with the authors’ knowledge and scoping review findings. The map was adjusted iteratively by discussion amongst the authors (MLO, JW, DN, and JD). Findings were fed back to all workshop participants for comment by email correspondence and face to face discussion, where practical; the map was further adjusted after this feedback.

### Ethical considerations

This priority setting workshop did not involve patients and did not use any personal identifying information. Ethical Review Board permission was therefore not required.

## Results

Thirty-four participants from different stakeholder groups attended the workshop. There was broad representation from professionals with knowledge and experience according to the different delays (“Appendix 1”). In brainstorming discussions, 42 barriers were generated across each delays. These barriers were subsequently assigned priorities of low (11/42), medium (12/42), and high (19/42) (Table 1).

**Table 1:** Identified barriers and their priority for further action

Delay	The barriers	Priority for further action
1	Religious beliefs/community decision making	High
1	General and health education/awareness	High
1	Perceived distance from health care	High
1	Poor recognition of injury severity	High
1	Preference for seeking traditional healer	High
1	Fear of loss of earnings	High
1	Domestic Violence and fear of reporting such	Medium
1	Difficulties in timely communication for those in society who are marginalized	Medium
1	Incomplete health insurance coverage	Low
1	Negative attitudes from previous experience, including prejudice	Low
1	Fear of the legal implications of assisting the injured	Low
1	Limited personal security at certain times/locations	Low
2	Inadequate number of available ambulances	High
2	Lack of ambulance fleet maintenance	High
2	Lack of private investment in ambulances	High
2	Inadequate ambulance equipment maintenance and stocking	High
2	Lack of public awareness of ambulance fees	High
2	Lack of central dispatch and precise geolocation of patients	Medium
2	Cost of capacity building	Medium
2	Cost to patient of transport	Medium
2	Poor quality of roads	Medium
2	Inadequate bystander awareness of responsibilities	Medium
2	Cost of accessing ambulances	Low
2	Lack of awareness of health service leaders	Low
2	Lack of knowledge on how to access the ambulance	Low
2	Inconsistent ambulance traffic priority	Low
3	Low referral trauma centre geographical coverage	High
3	Lack of protocols for bypass to referral centre	High
3	Non-commensurate number/location of trained personnel in hospitals	High
3	Unreliable availability of equipment in hospital	High
3	Inadequate facility infrastructure	High
3	Training and retention of specialist staff	High
3	Patchy trauma training expertize outside of referral centres	Medium
3	Inadequate insurance coverage	Low
3	Lack of training in use and maintenance of medical equipment	Low
4	Indirect cost of attending follow-up	High
4	Lack of resources for rehabilitation	High
4	Inequity	Medium
4	Lack of information of availability and need for services	Medium
4	Poor follow-up system	Medium
4	Poor services	Medium
4	Culture	Low

Barriers securing the majority vote after the first two rounds were; 1. “*Training and retention of specialist staff*”, 2. “*General and health education/awareness*”, and 3. “L*ow referral trauma centre geographical coverage*” (Table 2). To discriminate between the remaining 6 barriers, a third round of voting was undertaken. The barrier “*Lack of protocol for bypass to referral centre*” was selected.

**Table 2:**
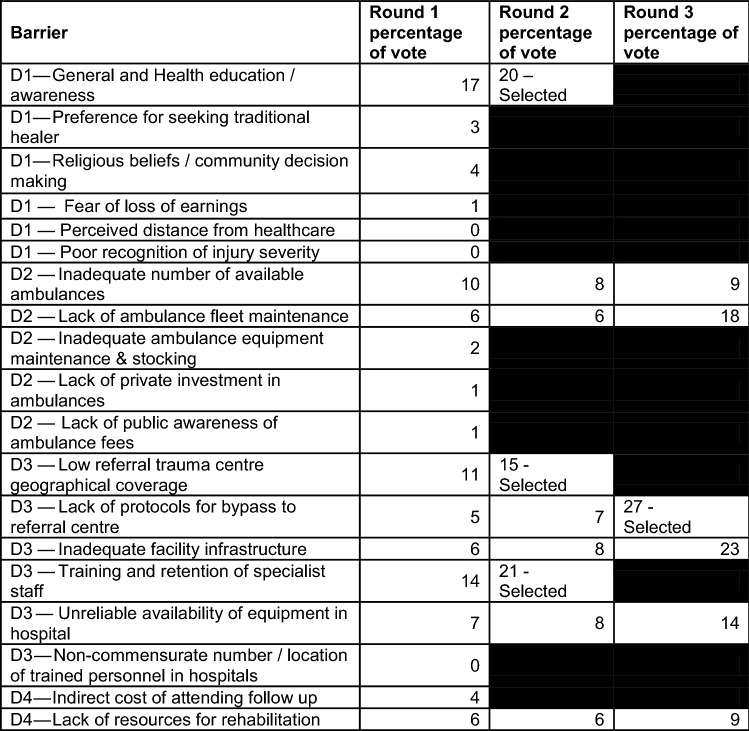
Results from the 3 round barrier prioritization exercise to identify the 4 most important barriers to injury care for further action

### Scoping review

The PubMed search identified 231 articles. Following title screening, 46 abstracts were identified as potentially relevant. Three duplicates were removed. Of the 43 unique abstracts, full text review identified 27 considered relevant to inform the understanding of barriers driving delays to injury or non-injury care within Rwanda. 16/27 articles directly studied injury whilst 11/27 were not injury related. 23/27 studies were from Rwanda only, whilst 4/27 incorporated other countries. Two studies reported an intervention, the remainder being observational. Both intervention studies were before and after studies; one evaluated the impact of delivering Advanced Trauma Life Support training on care process and patient outcome measures at a single centre [21]. Another reported a multi-centre multinational implementation of the WHO trauma care checklist for which 1/11 centres was based in Rwanda [22].

For 26/42 barriers to injury care identified in the stakeholder workshop, there was at least one published study which provided corroborating evidence of delays to access to care for injury (Table 3). Two barriers identified in our workshop had studies evidencing them delaying care for other health problems in Rwanda. Supporting evidence from the published literature was not found for 14 workshop identified barriers. Of 19 high-priority barriers, 14 were supported by at least one injury related publication including all four highest priority barriers. The remaining five high-priority barriers lacking published evidence were “*religious beliefs/community decision making*”, “*lack of ambulance fleet maintenance*”, “*inadequate ambulance equipment maintenance and stocking*”, “*lack of private investment in ambulances*” and “*lack of public awareness of ambulance fees*” (Table 3).

**Table 3:** Linking published evidence to proposed barriers to care

Delay	The barriers	Number of published studies reporting barrier	Study references	Participant priority (low, medium, high)	Rwanda barrier evidence volume^a^	
		Injury studies	Non-injury studies			
1	Incomplete health insurance coverage	3	4	Injury: Zafar et al. [23], Mpirimbanyi et al. [24], Petroze et al. [25] Non-injury: Roder-DeWan et al. [13], Musafili et al. [26], Lorent et al. [27], Ruktanonchai et al. [28]	Low	A
	Fear of loss of earnings	1	0	Injury: Matheson et al. [29]	High	B
	General and Health education/awareness	2	1	Injury: Mpirimbanyi et al. [24], Matheson et al. [29] Non-Injury: Roder-DeWan et al. [13]	High	A
	Perceived distance from health care	3	1	Injury: Mpirimbanyi et al. [24], Petroze et al. [25], Matheson et al. [29] Non-Injury: Ruktanonchai et al. [28]	High	A
	Poor recognition of injury severity	3	4	Injury: Mpirimbanyi et al. [24], Petroze et al. [25], Matheson et al. [29] Non-Injury: Roder-DeWan et al. [13], Umuhoza et al. [30], Musafili et al. [26], Pace et al. [31]	High	A
	Preference for seeking traditional healer	1	3	Injury: Mpirimbanyi et al. [24] Non-Injury: Roder-DeWan et al. [13], Umuhoza et al. [30], Pace et al. [31]	High	B
	Religious beliefs/community decision making	0	0		High	D
	Negative attitudes from previous experience and prejudice	1	1	Injury: Petroze et al. [25] Non-Injury: Roder-DeWan et al. [13]	Low	B
	Limited personal security at certain times/locations	0	0		Low	D
	Fear of the legal implications of assisting the injured	0	0		Low	D
	Domestic Violence and fear of reporting such	0	1	Non-Injury: Ntaganira et al. [32]	Medium	C
	Difficulties in timely communication for those in society who are marginalized	0	0		Medium	D
2	Poor quality of roads	1	2	Injury: Petroze et al. [25] Non-Injury: Niyitegeka et al. [33], Musafili et al. [26]	Medium	B
	Lack of central dispatch and precise geolocation of patients	0	0		Medium	
	Inadequate number of available ambulances	2	1	Injury: Mpirimbanyi et al. [24], Aluisio et al. [34] Non-Injury: Nkusi et al. [35]	High	A
	Lack of ambulance fleet maintenance	0	0		High	D
	Inadequate ambulance equipment maintenance & stocking	0	0		High	D
	Lack of private investment in ambulances	0	0		High	D
	Cost to patient of transport	2	3	Injury: Zafar et al. [23], Petroze et al. [25] Non-Injury: Roder-DeWan et al. [13], Musafili et al. [26], Bayitondere et al. [36]	Medium	A
	Cost of capacity building	0	0		Medium	D
	Cost of accessing ambulances	0	0		Low	D
	Lack of knowledge on how to access the ambulance	1	0	Injury: Petroze et al. [25]	Low	B
	Inconsistent ambulance traffic priority	0	0		Low	D
	Lack of awareness of health service leaders	0	0		Low	D
	Inadequate bystander awareness of responsibilities	1	0	Injury: Patel et al. [37]	Medium	B
	Lack of public awareness of ambulance fees	0	0		High	D
3	Low referral trauma centre geographical coverage	2	0	Injury: Krebs et al. [38], Mpirimbanyi et al. [24]	High	A
	Lack of protocols for bypass to referral centre	1	0	Injury: Mpirimbanyi et al. [24]	High	B
	Non-commensurate number/location of trained personnel in hospitals	3	1	Injury: Mpirimbanyi et al. [24], Chokotho et al. [39], Calland et al. [40] Non-injury: Tuyisenge et al. [41]	High	A
	Inadequate facility infrastructure	3	1	Injury: Mpirimbanyi et al. [24], Chokotho et al. [39], Nkurunziza et al. [42] Non-injury: Musafili et al. [26]	High	A
	Unreliable availability of equipment in hospital	3	1	Injury: Mpirimbanyi et al. [24], Chokotho et al. [39], Calland et al. [40] Non-injury: Musafili et al. [26]	High	A
	Inadequate insurance coverage	4	2	Injury: Mpirimbanyi et al. [24], Petroze et al. [25], Matheson et al. [29], Nkurunziza et al. [42]	Low	A
				Non-injury: Roder-DeWan et al. [13], Ruktanonchai et al. [28]		
	Patchy trauma training expertize outside of referral centres	5	1	Injury: Mpirimbanyi et al. [24], Petroze et al. [21], Calland et al. [40], Nkusi et al. [43], Lashoher et al. [22] Non-injury: Tuyisenge et al. [41]	Medium	A
	Lack of training in use and maintenance of medical equipment	0	0		Low	D
	Training and retention of specialist staff	4	1	Injury: Mpirimbanyi et al. [24], Calland et al. [40], Chokotho et al. [39], Ntakiyiruta et al. [44] Non-injury: Tuyisenge et al. [41]	High	A
4	Inequity	2	1	Injury: Aluisio et al. [34], Atijosan et al. [45] Non-Injury: Kikuchi et al. [46]	Medium	A
	Indirect cost of attending follow-up	1	1	Injury: Matheson et al. [29] Non-Injury: Bayitondere et al. [36]	High	B
	Culture	1	2	Injury: Matheson et al. [29] Non-Injury: Kikuchi et al. [46], Roder-DeWan et al. [13]	Low	B
	Lack of information of availability and need for services	1	0	Injury: Matheson et al. [29]	Medium	B
	Lack of resources for rehabilitation	1	0	Injury: Matheson et al. [29]	High	B
	Poor follow up system	0	1	Non-Injury: Roder-DeWan et al. [13]	Medium	C
	Poor services	1	2	Injury: Atijosan et al. [45] Non-Injury: Bayitondere et al. [36], Roder-DeWan et al. [13]	Medium	B

^a^Volume of evidence defined as: *A* ≥ 1 injury study describes the barrier, *B* = only 1 injury study describes the barrier, *C* = 0 injury study but 1 or more non-injury studies describe the barrier, *D* = 0 studies identified that describe the barrier

### Visualization of the barriers

**Fig. 1:**
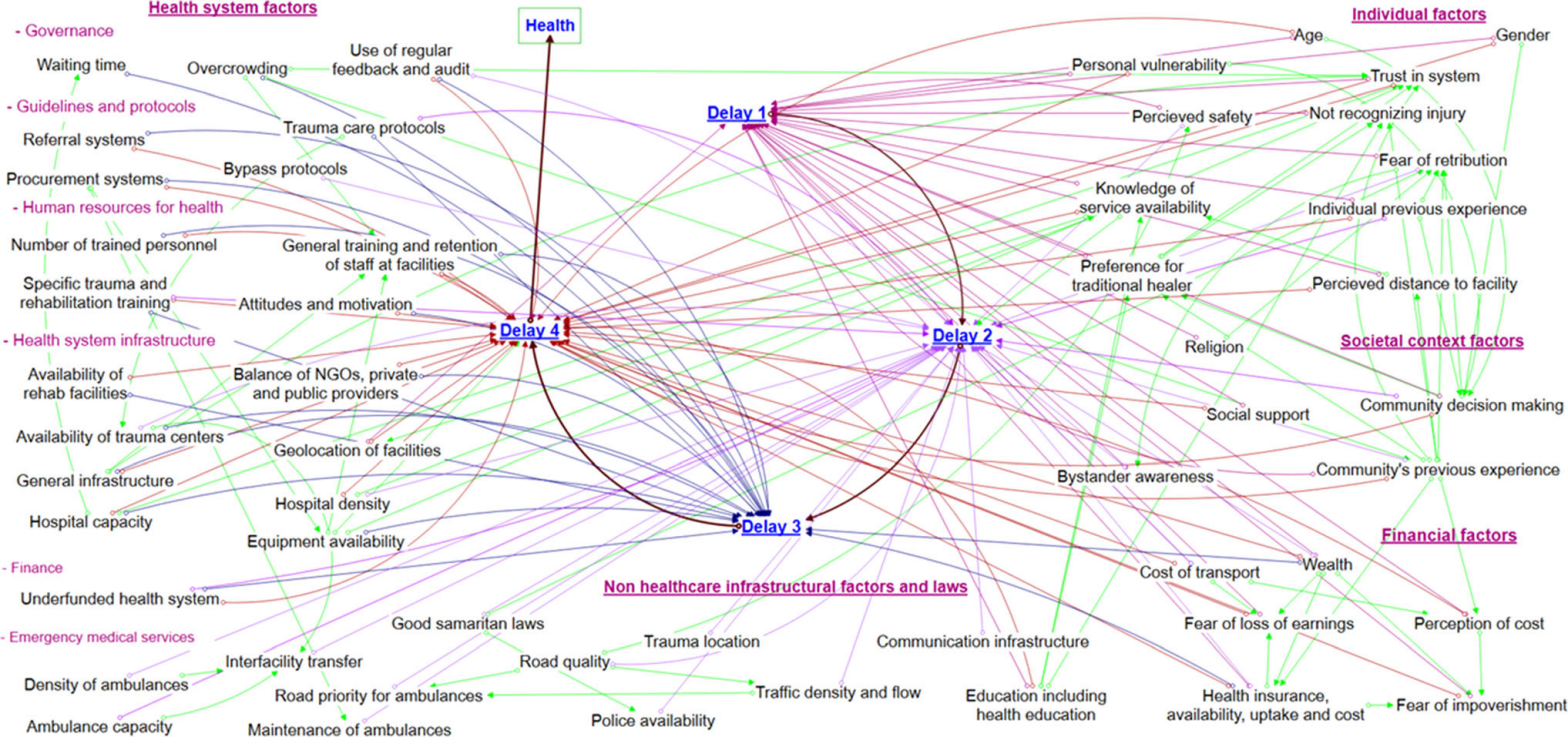
Visual representation of proposed barriers to injury care and their relationships to each conceptual delay

**Fig. 2:**
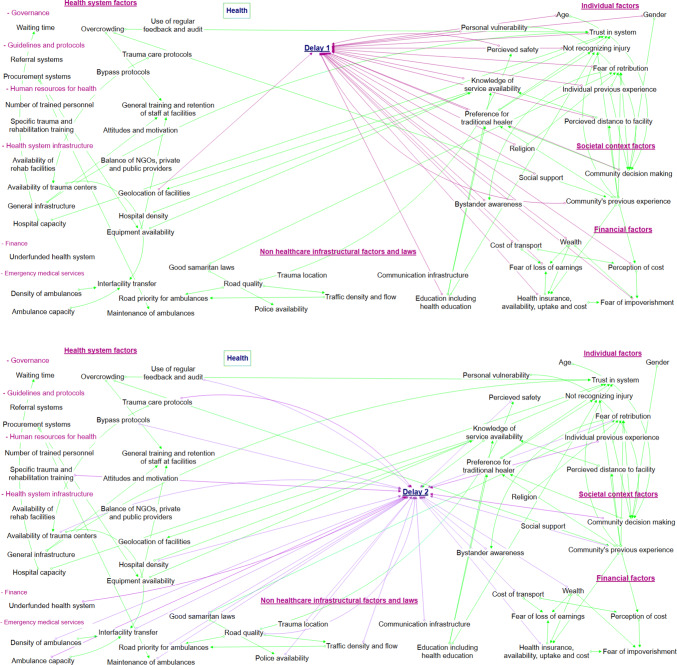

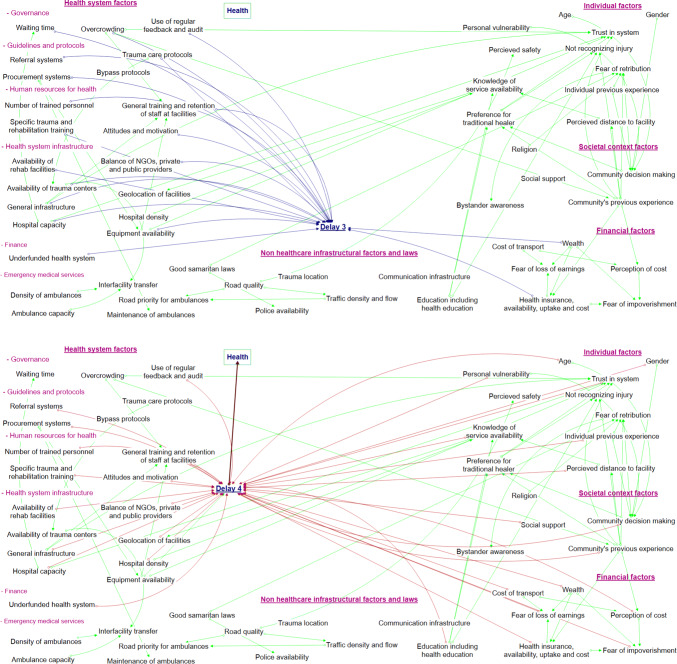

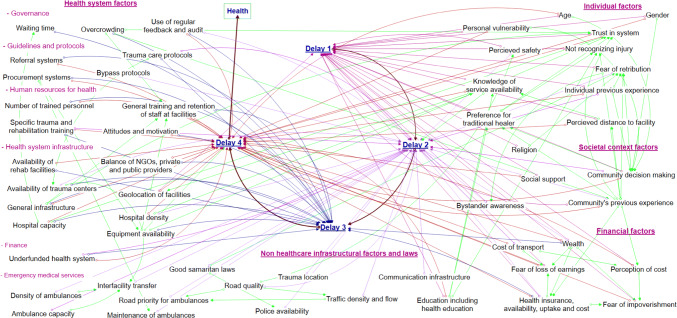
Visual representation of proposed barriers to injury care shown per conceptual delay

The barriers were divided into five overarching categories; individual factors, societal factors, financial factors, general infrastructural factors, and health-system infrastructural factors. More granular categories were avoided to ensure the visual representation was interpretable. Barriers at each delay and across all the delays combined are shown in Figs. 1 and 2. Iterative refining and revision of the barriers resulted in 54 barriers within these five categories. Some barriers are shown acting distinctly within just one delay whilst others impact across multiple. For example, “trauma location” is only linked to delay 2, whilst “health insurance availability, uptake and cost” was identified to have substantial impacts upon multiple delays (“Appendix 2”). The inter-relationships between barriers along with the theorized direction of impact is shown using arrows (Figs. 1 and 2).

## Discussion

This study is the first that we are aware of to identify all barriers to accessing injury care from the point of injury to being rehabilitated to maximal function in a low-income country, to visually represent their inter-relationships, prioritize them for future research and intervention, and identify which had been previously investigated in scientific studies. We utilized a four delay extension to the three delays framework, well established for assessing barriers to maternal, neonatal, and child health [47–51]. The three delays has shown utility to describe, classify and assess LMIC emergency and trauma systems [11, 12, 52]. The fourth delay has also been previously conceptualized as the delay in communities taking responsibility for avoidable mortality [53]. However, we preferred the definition of delay to remaining within the health care system [13]. By including it, our findings can inform rehabilitation service development in Rwanda, potentially benefiting 70,000 Rwandans living with injury-related musculoskeletal impairment, of whom almost half have not accessed adequate treatment [29].

Multiple barriers were identified across all delays in our study, falling under different (and sometimes multiple) overarching categories, inter-related with each other in a highly complex manner. Minimal research on interventions to address these barriers has been carried out in Rwanda, and identified studies mostly focused on tertiary facility-level care. The four highest priority barriers selected by workshop participants covered barriers impacting across all four delays.

There is a global health care workforce crisis, with workforce density particularly low in Sub-Saharan Africa [54, 55]. It is therefore understandable that the “*training and retention of specialist staff*” was given high priority for action by the workshop participants. International migration of health care workers is substantial. Over 40% Rwandan-born physicians practised in high-income countries in 2000 [56]. However, skilled health workforce density (physicians, nurses, and midwives) increased from 0.48 to 0.79 per 1000 population from 2005 to 2015 [57], though still considerably lower than higher income countries [58]. Workforce retention is likely particularly important in rural areas, where most Rwandans live [59, 60]. Emergency Medicine specialty training implemented in Kigali has shown mortality benefit at the University Teaching Hospital—Kigali [61]; the effects of such training programs in other locations needs to be investigated.

“*General and health education/awareness*” was a high-priority barrier not specifically concerning facility-level care. Zambian community members similarly identified improving emergency condition recognition and bystander first aid provision as important health-system intervention targets [62]. Health care literacy has similarly been found a barrier to LMIC injury care though Verbal Autopsy analysis and stakeholder Delphi studies [11, 12].

Most injury related procedures in University Teaching Hospital, Kigali, are for patients transferred from outside of Kigali [44]. “*Low referral trauma centre geographical coverage*” enabling provision of advanced trauma care has been shown to be sub-optimal elsewhere. The Lancet Commission on Global Surgery identified that 5 billion people, globally, lacked timely access to quality surgical care [9] including trauma treatment through emergency laparotomy and open fracture. In only 16 of 48 countries in sub-Saharan Africa, 80% of the population can access to public hospitals providing emergency care within 2 h [63]. However, such studies use geospatial mapping data that may not represent actual experienced travel time, especially in the rainy season [64].

“*Lack of protocols for bypass to referral centre*” to enable injury patients to be treated at the right hospital at the right time was the final barrier prioritized in our workshop. Developing bypass protocols can enable urgent cases to access more advanced injury care quickly, whilst limiting overburdening higher-level facilities with lower priority cases. This is recommended by the WHO as best practice for prehospital trauma care systems [65]. There is evidence from high-income countries showing lower risk of death for those transported directly to a Level 1 trauma centre [66, 67]. Although, comparable evidence from sub-Saharan Africa is lacking.

Health systems have been described as complex adaptive systems, nonlinear, counter-intuitive, and resistant to change [68]. Outside of trauma care, visual representations and interpretations of complex phenomena have been advocated to aid understanding such systems [69]. By visually representing the barriers and the associations between them within a four delays framework, our study can support researchers and policy makers understanding the complexity of Rwanda and other countries’ trauma care health systems and critically evaluating potential targets and consequences of interventions.

Our study has limitations. Only 34 participants were included and wider participation could have identified more barriers. Most participants were health care providers perhaps more inclined to prioritize barriers to receiving care. Patients or patient advocates were not included, missing their perspective or perceived priorities. Neither were police representatives included, often first to an injury scene. The schematic representation of the refined barriers was undertaken by the writing group members (MLO, JW, DN, and JD). Feedback from workshop participants was obtained, but the distant approach may have limited meaningful participation. Published evidence was scoped from one database and focused on Rwanda only. Expanding search terms, including additional databases and broadening geographic scope may yield additional corroborating evidence. However, an extensive systematic literature search was beyond the aims of this study.

This is the first workshop aiming to capture the complexity of barriers to access of quality injury care in Rwanda, and as far as we are aware, in any LMIC. Previous studies related to injuries in Rwanda have focused on disease burden and epidemiology, commonly related to road traffic collisions specifically. Although some groups were not represented in our workshop, we purposively invited people with research or work experience linked to each delay. Therefore, we trust the workshop captured most barriers linked to the different delays, and the richness and complexity of the data are clearly illustrated in the visual representation of barriers.

## Conclusion

In this study, we have identified, prioritized, and visually represented barriers in access injury care within Rwanda. These manifold barriers are complexly interconnected. Theoretically, therefore, addressing one of the highly prioritized barriers could impact positively on other barriers and delays. This theoretical understanding, along with stakeholder expressed priorities, can guide both researchers and policy makers alike in planning future research and interventions to improve injury care for the people of Rwanda and other LMICs.

## Appendix 1

See Table 4.

**Table 4:** Role, expertize, and country of primary workplace of the participants in the workshop

Profession/role	Expertize	Country of primary work	Number
Sociologist	Health seeking behaviour	UK	1
Prehospital care provider	Prehospital care	Rwanda	3
Anaesthesiologist	Prehospital care	Rwanda	1
Anaesthesiologist	Critical care	Rwanda	3
Surgeon	Surgical care	Rwanda	1
Surgeon	Writing Group/surgical care	Rwanda	2
Surgeon	Trauma care research	UK	1
Surgeon	Writing Group/health systems research	UK	1
Neurosurgeon	Neurosurgical care	Rwanda	1
Physician	Emergency care	Rwanda	2
Emergency Physician	Emergency Care	Rwanda	4
Gynaecologist	Health seeking behaviour	Rwanda	1
Paediatrician	Paediatric care and health seeking behaviour	Rwanda	1
Medical Doctor	Prehospital care	Rwanda	1
Medical Doctor	Writing Group/health systems research	UK	2
Medical Doctor	Red Cross NGO perspective	Rwanda	1
Medical Doctor, Public Health	NCD research	Rwanda	1
Global Health Fellow	Health systems research	Rwanda	1
Global Health Fellow	Health systems research	UK	1
Rwanda Social Security Board Staff	Health care financing	Rwanda	1
Computer engineering	Information and technology	Rwanda	1
Medical Student	Medical Student	Rwanda	1
“In Charge” of Injuries and disabilities at Rwanda Biomedical Centre	Injury Research	Rwanda	1
Physiotherapist	Physiotherapy and rehabilitation	Rwanda	1

## Appendix 2

See Table 5.

**Table 5:** Barriers as they appear in the visual representation, with overarching themes, and delays

The barriers	Linked to delay			
*Individual factors*				
Age	1			4
Gender	1			4
Trust in system	1			4
Not recognizing injury	1			
Perceived safety	1	2		
Personal vulnerability	1			4
Individual previous experience	1	2		4
Knowledge of service availability	1			4
Perceived distance to facility	1			4
Religion	1			
Preference for traditional healer	1			
Fear of retribution	1	2		
*Societal context factors*				
Social support	1			4
Community decision making	1			4
Community’s previous experience	1	2		4
Bystander awareness	1	2		
*Financial factors (personal)*				
Cost of transport		2		4
Wealth	1	2	3	4
Perception of cost	1			4
Fear of loss of earnings	1			4
Fear of impoverishment	1			4
Health insurance, availability, uptake, and cost	1	2	3	4
*Non-health care infrastructural factors and laws*				
Education including health education	1			4
Communication infrastructure		2		
Traffic density and flow		2		
Trauma location		2		
Police availability		2		
Road quality		2		
Good Samaritan laws		2		
**Health system factors**				
*Governance*				
Use of regular audit and feedback		2	3	4
Waiting time			3	
Overcrowding			3	
*Guidelines and protocols*				
Procurement systems			3	4
Bypass protocols		2		
Trauma care protocols		2	3	
Referral systems			3	4
*Human resources for health*				
Number of trained personnel			3	4
General training and retention of staff at facilities			3	4
Attitudes and motivation			3	4
Specific trauma training		2	3	4
*Health system infrastructure*				
Balance of NGOs, private and public providers			3	4
Availability of rehab facilities			3	4
Geolocation of facilities	1			4
Availability of trauma centres		2	3	
General infrastructure			3	4
Hospital density		2		4
Equipment availability			3	
Hospital capacity			3	4
*Finance*				
Underfunded health system				
*Emergency medical services*				
Ambulance capacity		2		
Density of ambulances		2		
Road priority for ambulances		2		
Maintenance of ambulance		2		
Interfacility transfer		2		
