# Correction to: Identifying, Prioritizing and Visually Mapping Barriers to Injury Care in Rwanda: A Multi-disciplinary Stakeholder Exercise

**DOI:** 10.1007/s00268-022-06584-z

**Published:** 2022-05-02

**Authors:** Maria Lisa Odland, John Whitaker, Dmitri Nepogodiev, Carolyn Achieng’ Aling’, Irene Bagahirwa, Theophile Dushime, Darius Erlangga, Christophe Mpirimbanyi, Severien Muneza, Menelas Nkeshimana, Martin Nyundo, Christian Umuhoza, Eric Uwitonze, Jill Steans, Alison Rushton, Antonio Belli, Jean Claude Byiringiro, Abebe Bekele, Justine Davies

**Affiliations:** 1 Institute of Applied Health Research, University of Birmingham, Birmingham, UK; 2 Faculty of Life Sciences and Medicine, King’s Centre for Global Health and Health Partnerships, King’s College London, Room 2.13, Global Health Offices, Weston Education Centre, Cutcombe Road, London, SE5 9RJ UK; 3 Academic Department of Military Surgery and Trauma, Royal Centre for Defence Medicine, Birmingham, UK; 4 National Institute for Health Research, Global Health Research Unit On Global Surgery, Institute of Translational Medicine, University of Birmingham, Birmingham, UK; 5 King Faisal Hospital, Kigali, Rwanda; 6 Rwanda Biomedical Centre, Kigali, Rwanda; 7 SAMU Division, Ministry of Health, Kigali, Rwanda; 8 Warwick Medical School, Population Evidence and Technologies, University of Warwick, Coventry, UK; 9 University of Rwanda College of Medicine and Health Sciences, Kigali, Rwanda; 10 University Teaching Hospital of Kigali, Kigali, Rwanda; 11 Department of Political Science and International Studies, School of Government and Society, University of Birmingham, Birmingham, UK; 12 School of Sport, Exercise and Rehabilitation Sciences, University of Birmingham, Birmingham, UK; 13 College of Medicine and Dental Sciences, NIHR Surgical Reconstruction and Microbiology Research Centre, University of Birmingham, Birmingham, UK; 14 University of Global Health Equity, Kigali, Rwanda; 15 Faculty of Health Sciences, Medical Research Council/Wits University Rural Public Health and Health Transitions Research Unit, University of Witwatersrand, Johannesburg, Gauteng South Africa

Correction to: World J Surg (2020) 44:2903–2918 https://doi.org/10.1007/s00268-020-05571-6

The following acknowledgment is missing from the original article:

The authors would like to acknowledge Jean Marie Uwitonze as an active contributor to this paper.

